# The efficacy and safety of pharmacologic thromboprophylaxis following caesarean section: A systematic review and meta-analysis

**DOI:** 10.1371/journal.pone.0208725

**Published:** 2018-12-10

**Authors:** Rui Yang, Xia Zhao, Yilei Yang, Xin Huang, Hongjian Li, Lequn Su

**Affiliations:** Pharmaceutical Department, Shandong Provincial Qianfoshan Hospital, Jinan,China; Medway NHS Foundation Trust, UNITED KINGDOM

## Abstract

**Objective:**

Our purpose is to evaluate the efficacy and safety of pharmacologic thromboprophylaxis following caesarean section (CS).

**Methods:**

We searched PubMed, Embase, and the Cochrane Library. Then the systematic review was performed by analysing studies that met the eligibility criteria.

**Results:**

Seven studies with 1243 participants were included, including 6 RCTs and 1 prospective cohort. Results from the meta-analysis showed that low molecular weight heparin (LMWH) was associated with no obvious decrease in the risk of thrombus compared with UHF and negative control. However, LMWH was observed to be associated with a definite increase in the risk of bleeding or haematomas in comparison to negative control (RR: 8.47, CI: 1.52–47.11).

**Conclusion:**

According to current evidences, the efficacy of pharmacologic thromboprophylaxis which increases the risk of bleeding or hematomas remains controversial.

## Introduction

Caesarean section (CS) rates have been dramatically increasing over the past decades worldwide[1]. In the United States, more than 30% of pregnant women gave birth in the form of CS in 2006[2], while 46.2% of Chinese new-borns, the highest percentage in Asia, were delivered by CS in 2010[3]. Deep vein thrombosis (DVT), a blood clot in a deep vein of the leg or lower pelvis, is one of the most serious complications after CS. DVT causes lower limb dysfunction, thrombosis syndrome, and fatal pulmonary embolism (PE) in many serious cases, which is a leading cause of maternal morbidity and mortality. The incidence of DVT following CS is approximately 0.5%, and 0.5% to 2.0% of patients with DVT will suffer life-threatening PE[4,5].

Given the serious consequences of DVT following CS, many mechanical preventive treatment strategies are often used in the postpartum period including early ambulation after surgery, graduated compression stockings, intermittent pneumatic compression, and others. Because of the lack of evidences, the benefits of pharmacologic thromboprophylaxis in preventing the occurrence of DVT in CS patients remain controversial. Guidelines from major societies, such as the Royal College of Obstetricians and Gynaecologists (RCOG), the American Congress of Obstetricians and Gynecologists (ACOG), and the American College of Chest Physicians (Chest), differ markedly in terms of criteria for identifying CS patients who should receive pharmacologic thromboprophylaxis[6,7]; the guidelines are mainly based on expert opinions rather than evidence-based medicine from randomized or other clinical trials[8]. Therefore, it is important and necessary to evaluate the efficacy of pharmacologic thromboprophylaxis following CS. This systematic review based on available clinical trials to compare different thromboprophylaxis outcomes was performed.

## Methods

### Inclusion and exclusion criteria

Studies were included if they met both of the following criteria: 1) patients were treated with pharmacological thromboprophylaxis for preventing DVT following CS; and 2) articles were published in English.

### Data sources and search strategy

The search proceeded in PubMed, Embase and the Cochrane Library, including keyword and free word searches. The following search keywords were used: ‘thrombophilia’, ‘thromboembolism’, ‘thromboprophylaxis’, ‘deep vein thrombosis’, ‘enoxaparin’, ‘heparin’, ‘caesarean section’, ‘Caesarean section’, ‘uterine-incision delivery’, ‘caesarean delivery’, ‘abdominal caesarean section’. In addition, this search was restricted to human trials with the final date of December 2017. The references of eligible articles were examined to filter further suitable articles. Endnote was used to remove duplicates and manage all references.

### Study selection and data extraction

During the screening process, all articles were assessed by title and abstract based on the eligibility criteria. All potentially eligible studies were evaluated by reading full texts, and the studies that met the eligibility criteria were included in our systematic review. Two researchers independently extracted the following information from included studies: I) General characteristics of patients: age, weight and sample size. II) Intervention: type of thromboprophylaxis agent, dosage, duration, comparator including placebo, other agents, or no treatment. III) Outcomes: number of thrombosis-induced death/DVT/PE, major bleeding events and other adverse events. All processes were carried out separately by two researchers, and all disagreements were handled by discussion or consulting a third-party researcher.

### Quality assessment

The methodological quality of RCTs was evaluated independently by two researchers using the Cochrane Collaboration’s tool[9,10]. For each of the seven domains, the study was ranked as high risk of bias, low risk of bias, or unclear risk of bias. The Newcastle-Ottawa Scale (NOS) was used to assess the bias risk of cohort studies with three factors including patient selection, comparability of groups, and outcome assessment. Studies were graded on an ordinal star scoring scale, with higher scores representing higher quality. The quality was ranked as high if it achieved 7 stars out of 9 points[11].

### Statistical analysis

Statistical analysis was conducted to estimate the Risk Ratios (RR) with 95% confidence intervals (CI) and to generate forest plots along with the heterogeneity assessment. The heterogeneity among studies was examined by the I-square (I^2^) statistic. If the I^2^ value was below 50%, a high degree of homogeneity was considered to exist among the studies. In this case, a fixed effects model was usedto replace a random effects model to estimate the RR[12].

## Results

### Study selection

**Fig 1:**
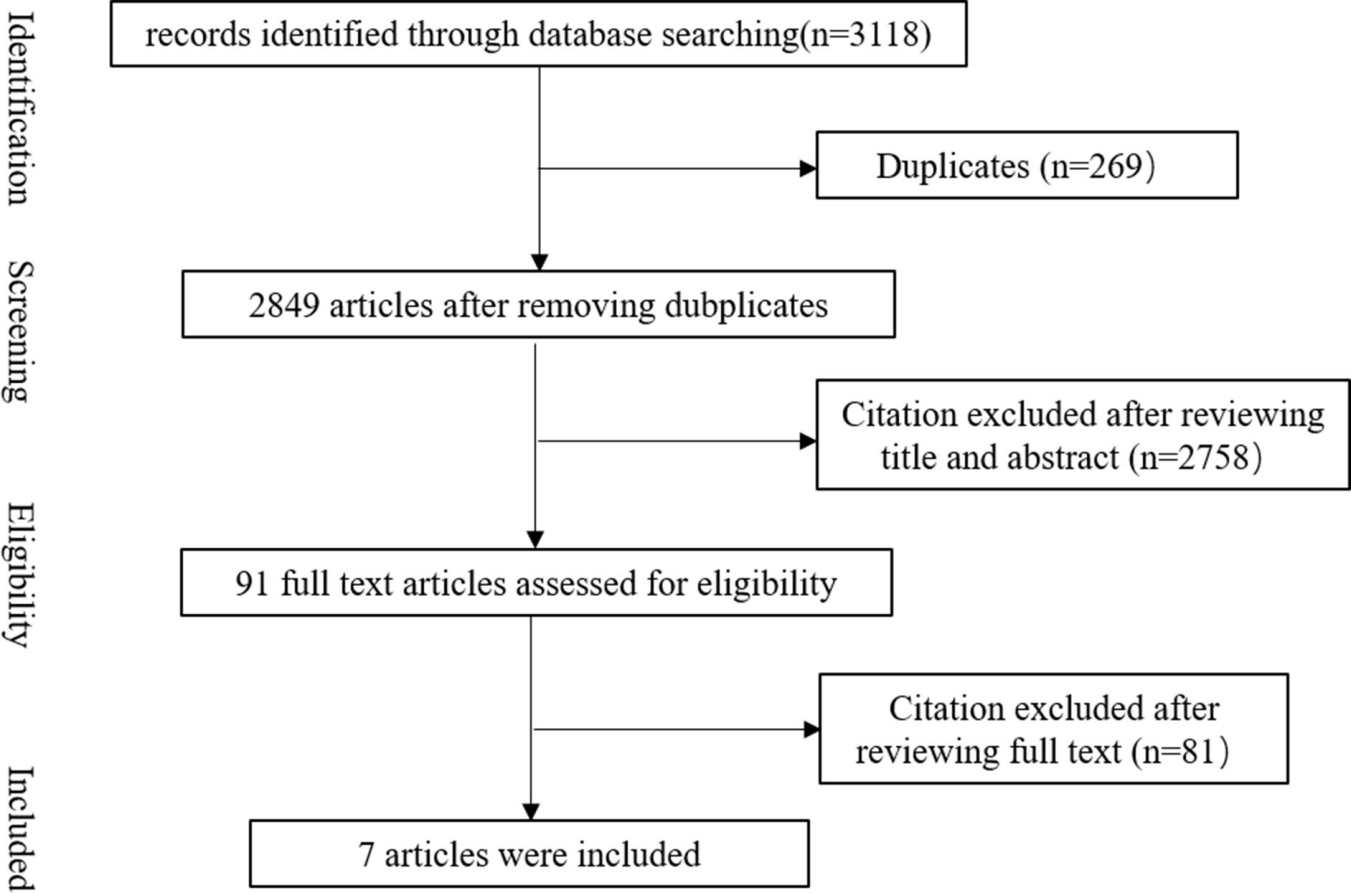
Flow chart of selection process.

A total of 3118 articles were identified through the included databases. After removing 269 duplicates, 2849 articles were obtained by initial screening. A total of 2758 of 2849 articles in the initial screening were excluded by screening titles and abstracts. The remaining 91 articles were reviewed by reading the full text. As a result, 7 articles were enrolled in this systematic review (Fig 1).

### Study characteristics

Six RCTs[13–18] and one cohort study[19], published from 1998 to 2014, were included in this systematic review. Three studies were conducted in the United Kingdom[14,16,17], and the other four studies were from Australia[13], K.S.A.[15], Germany[18], and Italy[19]. This meta-analysis included a total of 1243 participants, with the sample size ranging from 17 to 529 cases. There were just two studies that distinguished patients with one or more risk factor of VTE following CS[14,17], such as obesity, immobility, maternal age > 35 years, parity > 4, labour > 12 h, gross varicose veins, current infection, pre-eclampsia, major current illness and CS performed as an emergency procedure.

Five studies[13,15,16,18,19] assessed the thromboprophylaxis efficacy of LMWH versus placebo, and two[17,18] research studies evaluated the efficacy between LMWH and UFH. Additionally, differences in LMWH were compared by different product[14] and dose[17]. The duration of the interventions differed among the included 7 studies, ranging from 5 to 14 days (Table 1).

**Table 1:** General characteristics of the enrolled studies.

Author	Year	Country	Study design	Number	Intervention		Age		Weight / BMI		Duration of prevention
					Treatment	Comparator	Treatment	Comparator	Treatment	Comparator	
Burrows RF[13]	2001	Australia	RCT	76	dalteparin 2500 IU (n = 39)	Saline (n = 37)	31.7±4.8	31.3±5.5	81.7[17.2]	79.9[14.0]	5 days
Ellison J[14]	2001	United Kingdom	RCT	30*	enoxaparin 4000 IU (n = 10) tinzaparin 50 IU/kg (n = 10) dalteparin 5000 IU (n = 10)	NR	26(18–35) 27(16–42) 28(16–40)	NR	BMI 28.2(22–41) 29.5(21–40.7) 27.8(23–39)	NR	5 days
Farjah A[15]	2012	K.S.A.	RCT	300	tinzaparin 4500 IU (n = 100)	Placebo (n = 200)	28.6 (18–35)	28.6 (18–35)	NR	NR	14 days
Gates S[16]	2004	United Kingdom	RCT	141	enoxaparin 40 mg (n = 70)	saline (n = 71)	31.3±5.8	30.6±5.4	≥80 kg, 29%	≥80 kg, 30%	14 days
Gibson J L[17]	1998	United Kingdom	RCT	17*	enoxaparin 20 mg (n = 6) enoxaparin 40 mg (n = 5)	UFH 7500IU ×2 (n = 6)	NR	NR	NR	NR	NR
Gizzo S[19]	2014	Italy	Prospective Cohort	529	enoxaparin 4000 UI or dalteparin 5000 UI (n = 349)	no treatment (n = 180)	38.07±2.58	38.3±2.77	BMI 27.14±2.16	BMI 27.48±1.93	7 days
Heilmann L[18]	2007	Germany	RCT	150	dalteparin 5000 U (n = 50) UFH 5000 IU×2 (n = 50)	no treatment (n = 50)	28±6 29±5	28±3	BMI 23±4 23±2	BMI 20±7	7 days

Abbreviations: *, in addition to CS, there was at least one additional risk factor for thrombosis

### Quality assessment

**Fig 2:**
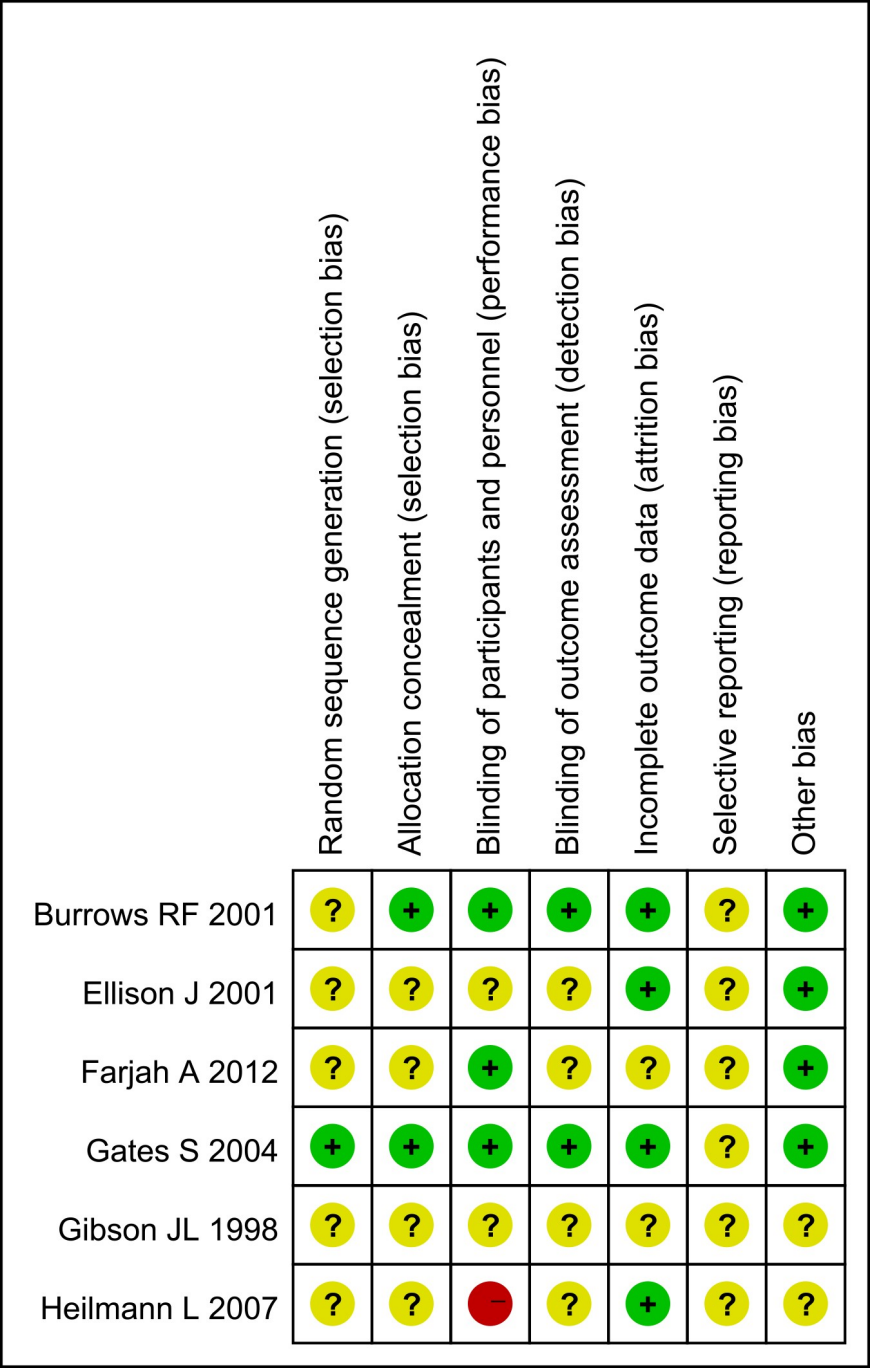
Risk of bias graph for 6 RCTs.

In the six included RCTs, five studies in Fig 2 were assessed to present an unclear risk of bias[13–17], while the sixth study was at high risk of bias[18]. In this high risk study, patients of the control group received no treatment, and failed blinding of participants and personnel was found. One cohort was determined as high quality: 8 stars[19].

### Efficacy evaluation

Because of the different comparators, the included studies were assigned to three subgroups: LMWH versus negative control, LMWH versus UFH, and LMWH versus LMWH. In the first subgroup, analysis of all 5 studies[13,15,16,18,19] showed that LMWH and the negative control were associated with no obvious decrease in the risk of VTE, PE, or death related to VTE [1.18, 95% *CI* = (0.28,4.91), *P* = 0.82]. The observed *I*^*2*^ of 0% showed that there was no heterogeneity among these studies.

**Fig 3:**
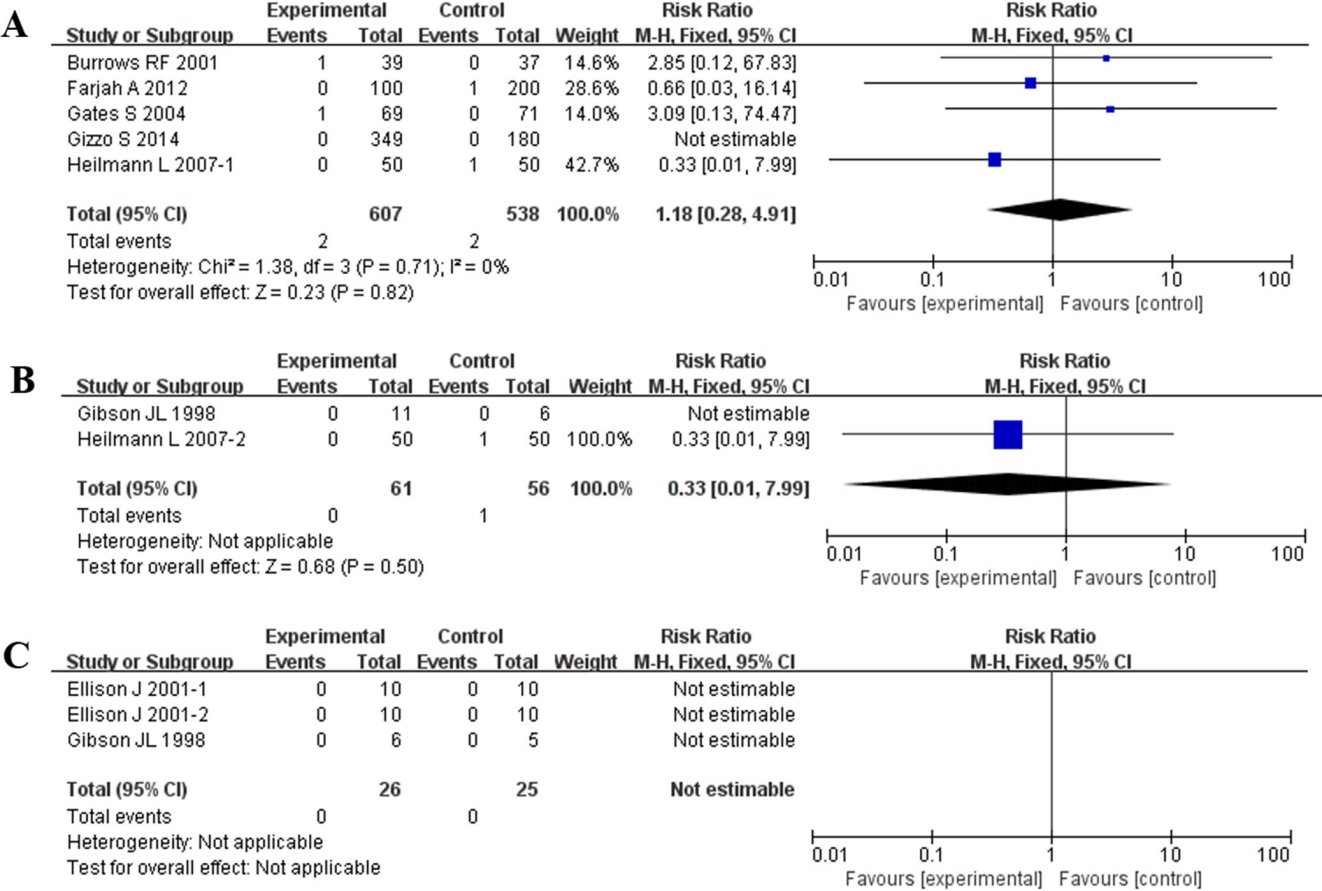
Effect of all studies in reducing the incidence of embolism. (A) LMWH versus negative control; (B) LMWH versus UFH; (C) LMWH versus LMWH.

In the second subgroup, two studies[17,18] including a total of 117 patients compared thromboprophylaxis of LMWH versus UFH. The outcomes of DVT and PE showed that the reduction of embolism was not significantly different [0.33, 95% *CI* = (0.01,7.99), *P* = 0.50] (Table 2, Fig 3).

There were two studies[14,17] involved in the last subgroup with only 41 patients, and the outcomes showed that no thrombosis occurred among all groups, such as those treated with enoxaparin, tinzaparin, and dalteparin.

**Table 2:** Summary of the meta-analysis of thromboprophylaxis efficacy following CS.

Subgroup	Included studies	N	RR (95% CI)	*I*^*2*^ (%)	P (%)
LMWH versus negative control	Overall	5	1.18(0.28–4.91)	0	0.82
	RCTs	4	1.18(0.28–4.91)	0	0.82
	Cohort	1	NE	NA	NA
	Unknown risk of VTE	5	1.18(0.28–4.91)	0	0.82
LMWH versus UFH	Overall	2	0.33(0.01–7.99)	NA	0.50
	RCTs	1	0.33(0.01–7.99)	NA	0.50
	Unknown risk of VTE	1	0.33(0.01–7.99)	NA	0.50
	High risk of VTE	1	NE	NA	NA
LMWH versus LMWH	Overall	3	NE	NA	NA
	RCTs	3	NE	NA	NA
	High risk of VTE	3	NE	NA	NA

Abbreviations: N, number of studies; RR, risk ratios; CI, confidence interval; P, P value for association; NE, not estimable; NA, not applicable.

### Safety evaluation

The reported adverse events in the 7 studies primarily included bleeding or haematomas, blood transfusions, allergic reactions, and serious wound complications such as wound infections requiring antibiotics, dehiscence, secondary sutures, and other treatment. All studies were assigned to the same subgroups based on a homologous comparator in efficacy evaluation.

**Fig 4:**
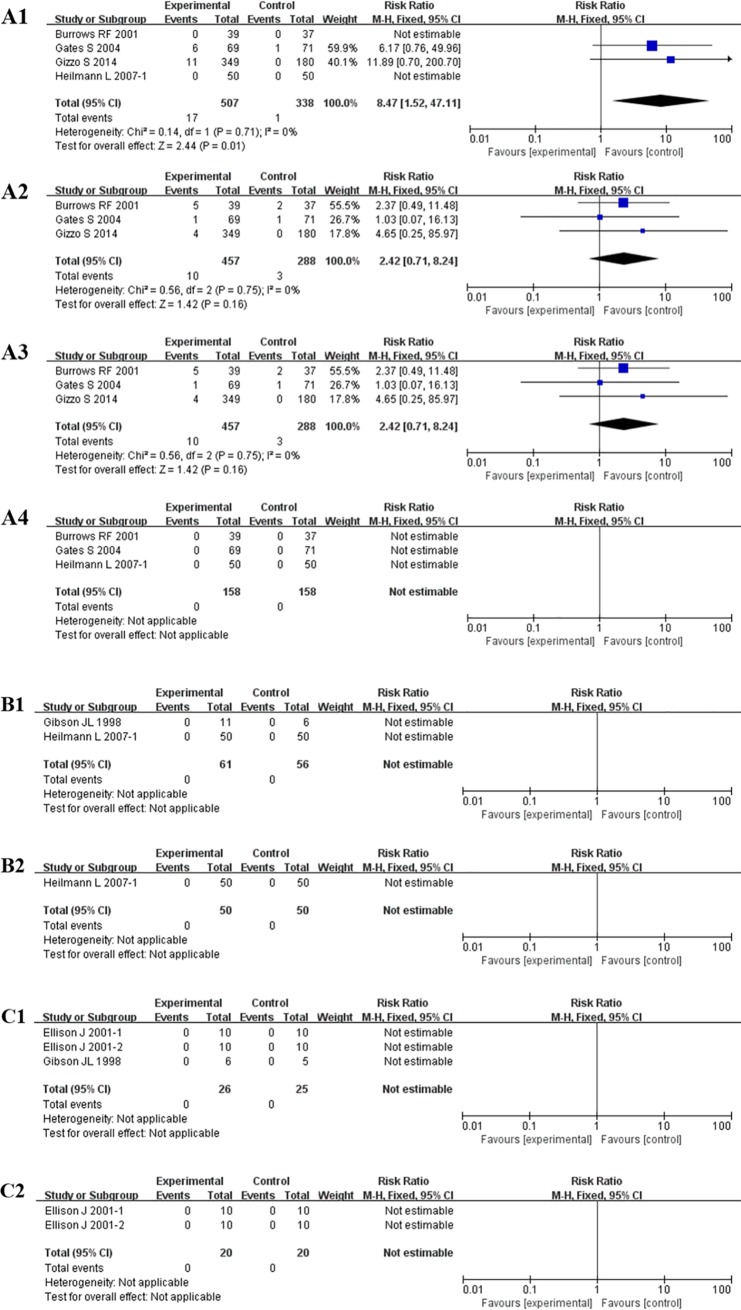
Safety of thromboprophylaxis following CS. A: LMWH versus negative control: (A1) Bleeding or hematomas; (A2) blood transfusion; (A3) wound complications; (A4) allergic reactions; B: LMWH versus UFH: (B1) Bleeding or hematomas; (B2) allergic reactions; C: LMWH versus LMWH: (C1) Bleeding or hematomas; (C2) allergic reactions.

In the first subgroup, the results of 4 studies[13,16,18,19] showed that LMWH and the negative control were associated with an obvious increase in the risk of bleeding or haematomas [8.47, 95% CI = (1.52, 47.11), *P* = 0.01]. The highest relative risk increment of 11.89 times was observed in the study of Gizzo S[19], while the highest weight coefficient of 59.9% was found in Gate S[16]. The observed *I*^*2*^ of 0% showed that there was no heterogeneity among these studies in the risk of bleeding or haematomas. For other adverse events including blood transfusion, wound complications and allergic reactions, the incidences showed no significant differences. In the second subgroup of LMWH versus UFH, the risk of bleeding or haematomas was compared in two studies[17,18] including a total of 117 patients: the result was not estimable, and the same conclusion was found regarding allergic reactions. For the last subgroup, the outcomes of occurrence rates of bleeding, haematomas or allergic reactions showed no significant differences among different products of LMWH (Table 3, Fig 4).

**Table 3:** Summary of the meta-analysis of thromboprophylaxis safety following CS.

subgroup	Included studies	N	Bleeding/ haematomas			N	Blood transfusion			N	Wound complications				N	Allergic reactions		
			RR (95% CI)	*I*^*2*^	P(%)		RR (95% CI)	*I*^*2*^	P(%)			RR (95% CI)	*I*^*2*^	P(%)		RR (95% CI)	*I*^*2*^	P(%)
LMWH versus negative control	overall	4	8.47 (1.52–47.11)	0	0.01	3	2.48(0.04–146)	73	0.66	3		2.42(0.71–8. 24)	0	0.16	3	NE	NA	NA
	RCTs	3	6.17(0.76–49.96)	NA	0.09	2	0.32(0.01–7.54)	NA	0.48	2		1.94(0.50–7.44)	0	0.34	3	NE	NA	NA
	Cohort	1	11.89(0.70–200.7)	NA	0.09	1	17.07(1.03–282)	NA	0.05	1		4.65(0.25–85.97)	NA	0.30	0	—	—	—
	Unknown risk of VTE	4	8.47 (1.52–47.11)	0	0.01	3	2.48(0.04–146)	73	0.66	3		2.42(0.71–8. 24)	0	0.16	3	NE	NA	NA
LMWH versus UFH	overall	2	NE	NA	NA	0	—	—	—	0		—	—	—	1	NE	NA	NA
	RCTs	2	NE	NA	NA	0	—	—	—	0		—	—	—	1	NE	NA	NA
	Unknown risk of VTE	1	NE	NA	NA	0	—	—	—	0		—	—	—	1	NE	NA	NA
	high risk of VTE	1	NE	NA	NA	0	—	—	—	0		—	—	—	0	—	—	—
LMWH versus LMWH	overall	3	NE	NA	NA	0	—	—	—	0		—	—	—	2	NE	NA	NA
	RCTs	3	NE	NA	NA	0	—	—	—	0		—	—	—	2	NE	NA	NA
	high risk of VTE	3	NE	NA	NA	0	—	—	—	0		—	—	—	2	NE	NA	NA

Abbreviations: N, number of studies; OR, odds ratio; CI, confidence interval; P, P value for association; NE, not estimable; NA, not applicable

## Discussion

Presently, the rate of CS, a significant risk factor for venous thromboembolism, has been steadily increasing over the past decades[1]. After CS, the incidences of PE and DVT are 0.06% and 0.04%, respectively, among the samples of the general population in Japan, which represent 22- and five-times higher risks than those after vaginal delivery[20,21]. Based on the serious risk of thrombosis, post-caesarean thromboprophylaxis has been advocated even though the evidence is still limited. Mechanical thromboprophylaxis, such as elastic stockings (ES) or intermittent pneumatic compression (IPC), are recommended[8]. Due to a lack of evidence from appropriately sized randomized trials, pharmacological thromboprophylaxis such as heparin is controversial. Different guidelines include inconsistent recommendations regarding thromboprophylaxis for women undergoing CS. For instance, both the Royal College of Obstetricians and Gynaecologists (RCOG) [22] and the Society of Obstetric Medicine of Australia and New Zealand (SOMANZ)[23] recommend heparin thromboprophylaxis for all emergency CS, while the American College of Chest Physicians[24] suggests heparin only in the presence of another co-existing risk factor, such as excessive body mass index (BMI). Therefore, the absence of evidence leads to a wide variation among different guidelines on prophylactic strategies following CS.

In this systematic review, we evaluated the thromboprophylaxis efficacy following CS based on the latest clinical research. The efficacy results showed that there were no statistically significant differences in the risk of thrombus among LMWH versus negative control, LMWH versus UHF and LMWH versus LMWH, with all 7 related studies including RCTs and cohort. In the safety evaluation, LMWH was observed to increase the risk of bleeding or haematomas by 8.47 times compared with placebo, while other indicators such as blood transfusion, wound complications and allergic reactions showed no significant differences. In the other two subgroups, including LMWH versus UFH and LMWH versus LMWH, the incidences showed no significant differences.

There were several limitations in this systematic review. Most of the included RCTs, many of which included a small sample size such as 17 enrolled cases, were conducted in a single centre. In view of the low incidence of thrombotic events, larger samples are required for further validation. Moreover, the quality of the RCTs should be improved with regard to random sequence generation, allocation concealment, and blinding of participants and personnel.

## Conclusions

Taken together, our studies indicate that there is insufficient evidence on which to base recommendations for pharmacologic thromboprophylaxis following CS. Large-scale, high-quality trials are warranted to evaluate the effectiveness of intervention.

## Supporting information

S1 PRISMA Checklist (DOC)
